# Optimizing Dose and Timing in Magnetic Tracer Techniques for Sentinel Lymph Node Detection in Early Breast Cancers: The Prospective Multicenter SentiDose Trial

**DOI:** 10.3390/cancers13040693

**Published:** 2021-02-09

**Authors:** Abdi-Fatah Hersi, Lida Pistiolis, Carlos Dussan Luberth, Eva Vikhe-Patil, Fredrik Nilsson, Imad Mohammed, Roger Olofsson Bagge, Fredrik Wärnberg, Staffan Eriksson, Andreas Karakatsanis

**Affiliations:** 1 Centre for Clinical Research, Region Västmanland—Uppsala University, Sigtunagatan, 72189 Västerås, Sweden; staffan.eriksson@regionvastmanland.se; 2 Department of Surgery, Västmanlands Hospital, Sigtunagatan, 72189 Västerås, Sweden; 3 Department of Surgery, Institute of Clinical Sciences, Sahlgrenska Academy at University of Gothenburg, Sahlgrenska University Hospital, 41345 Gothenburg, Sweden; lida.pistioli@vgregion.se (L.P.); roger.olofsson.bagge@vgregion.se (R.O.B.); fredrik.warnberg@surgsci.uu.se (F.W.); 4 Department of Surgery, Linköping University Hospital, 58185 Linköping, Sweden; carlos.dussan.luberth@regionostergotland.se (C.D.L.); eva.vikhe.patil@regionostergotland.se (E.V.-P.); 5 Department of Surgery and Perioperative Sciences, Umeå University Hospital, 90187 Umeå, Sweden; fredrik.nilsson@umu.se; 6 Department of Surgery, Kalmar County Hospital, 39185 Kalmar, Sweden; imad.mohammed@ltkalmar.se; 7 Department of Surgical Sciences, Uppsala University, Akademiska Sjukhuset, 75185 Uppsala, Sweden; andreas.karakatsanis@surgsci.uu.se

**Keywords:** sentinel lymph node biopsy, breast cancer, superparamagnetic iron oxide, magnetic tracer, sentinel lymph node

## Abstract

**Simple Summary:**

Superparamagnetic iron oxide (SPIO) nanoparticles have comparable performance to the combination of radioisotope and blue dye (RI + BD) for sentinel lymph node (SLN) biopsy in breast cancer. In this multicenter prospective study, lower SPIO doses (undiluted 1.5 vs. 1.0 mL) in different timeframes (perioperative vs. 1–7 days preoperative) and injection sites (subareolar vs. peritumoral) were compared to the previous standard (diluted 2.0 mL perioperatively) from the earlier Nordic trial. RI + BD were co-administered as background. In total, 534 patients were analyzed. SPIO SLN detection rates were similar (97.5% vs. 100% vs. 97.6%, *p* = 0.11) and respectively non-inferior to the dual technique. Significantly more SLNs were retrieved in the preoperative 1.0 mL cohort compared with 1.5 mL and the Nordic cohorts (2.18 vs. 1.85 vs. 1.83, *p* = 0.003). Thus, SPIO at 1.5 and 1.0 mL was non-inferior to both Sienna+^®^ and the dual technique for SLN detection.

**Abstract:**

Superparamagnetic iron oxide nanoparticles (SPIO) are non-inferior to radioisotope and blue dye (RI + BD) for sentinel lymph node (SLN) detection. Previously, 2 mL SPIO (Sienna+^®^) in 3 mL NaCl was used. In this dose-optimizing study, lower doses of a new refined SPIO solution (Magtrace^®^) (1.5 vs. 1.0 mL) were tested in different timeframes (0–24 h perioperative vs. 1–7 days preoperative) and injections sites (subareolar vs. peritumoral). Two consecutive breast cancer cohorts (*n* = 328) scheduled for SLN-biopsy were included from 2017 to 2019. All patients received isotope ± blue dye as back-up. SLNs were identified primarily with the SentiMag^®^ probe and thereafter a gamma-probe. The primary endpoint was SLN detection rate with SPIO. Analyses were performed as a one-step individual patient-level meta-analysis using patient-level data from the previously published Nordic Trial (*n* = 206) as a third, reference cohort. In 534 patients, the SPIO SLN detection rates were similar (97.5% vs. 100% vs. 97.6%, *p* = 0.11) and non-inferior to the dual technique. Significantly more SLNs were retrieved in the preoperative 1.0 mL cohort compared with 1.5 and the 2.0 mL cohorts (2.18 vs. 1.85 vs. 1.83, *p* = 0.003). Lower SPIO volumes injected up to 7 days before the operation have comparable efficacy to standard SPIO dose and RI + BD for SLN detection.

## 1. Introduction

Sentinel lymph node biopsy (SLNB) is the standard axillary staging method in patients with breast cancer without clinically evident nodal spread [1] and is associated with similar oncologic outcomes but less morbidity than conventional axillary lymph node dissection (ALND) [2,3,4,5]. Traditionally, detection with a radioisotope tracer (RI) combined with blue dye (BD), with a detection rate of more than 95%, has been regarded gold standard [1,6,7,8]. This method of combining tracers is known as the “dual technique”. However, several drawbacks such as limited access, rigid legislation on radioactive disposal, short half-life of RI [9] as well as anaphylactic reactions and skin staining at the injection site related to the use of BD [10,11] limit its usage.

Superparamagnetic iron oxide nanoparticles (SPIO) are a SLNB tracer with comparable detection rates as the dual technique but provide logistical advantages such as increased flexibility in the timeframe of administration [12,13,14]. An earlier version (Sienna+^®^, Endomagnetics Ltd., Cambridge, UK) required dilution (2 mL SPIO + 3 mL NaCl 0.9%). Adverse effects included patient discomfort, artifacts on magnetic resonance imaging (MRI) and brown skin staining [15,16]. Previous reports indicated higher detection rates if SPIO was injected 1–28 days before surgery, instead of on the day of surgery [14,17,18]. Recently, a new solution of SPIO (Magtrace^®^, 2 mL, Endomagnetics Ltd.) with no need for dilution, has been shown to be noninferior to the dual technique [19,20].

The aim of this study was to compare the SLN detection rate using Magtrace^®^ at lower doses, with different timeframes and injection sites, and to investigate whether they were noninferior to the previous SPIO solution of Sienna+^®^.

## 2. Methods

This multicenter prospective trial enrolled patients scheduled for primary breast surgery including SLNB at six Swedish centers. Inclusion criteria were breast cancers graded cT_0–2_cN_0_cM_0_, and Eastern Cooperative Oncology Group (ECOG) performance status 0–2. All patients provided oral and written consent. Patients with previous ipsilateral breast or axillary surgery and/or radiation and neoadjuvant chemotherapy were excluded. The dataset of the Nordic SentiMag trial [13] was used to derive reference values and for subsequent patient-level comparisons. The study was approved by the Uppsala University regional ethics committee (Decision Number 2017/063), registered in a prospective database (ISRTCN11156955) and monitored by an independent external agency.

### 2.1. Procedure

Magtrace^®^ was administered in two different sequential settings: the first patient cohort received a periareolar injection of 1.5 mL SPIO on the day of surgery, not later than 20 min prior to the start of surgery, followed by a five minute massage. The second patient cohort received 1.0 mL SPIO by subareolar or peritumoral injection into the interstitial tissue without massage, 1–7 days before surgery. All patients received RI and BD, according to routine practice.

During surgery, the surgeon initially used the Sentimag^®^ (Endomagnetics Ltd.) to localize the SLN and then used the gamma probe to confirm this, both before and after skin incision. All SLNs detected intraoperatively with the Sentimag^®^, gamma probe or stained brown or blue were excised. The conventional cut-off of 10% of the SLN with the highest signal (SPIO or RI) was implemented. After excision, ex vivo counts for each lymph node with both probes were registered. SLN status was assessed by routine histopathology.

### 2.2. Sample Size Calculation, Statistical Analysis and Data Collection

The main objective was to evaluate whether administration of Magtrace^®^ as described above was non-inferior to Sienna+^®^ for SLN detection. We used the earlier detection rate of 97% with Sienna+^®^ from the Nordic trial [13] and defined a non-inferiority margin of 4%, resulting in a lower threshold of 93%, to declare non-inferiority. For this, a sample size of 150 per cohort with a minimum of 146 successful magnetic SLNB procedures was required, to ensure that the lower 95% confidence interval of the detection rate proportion would still be >93%. Allowing for a 10% dropout rate, 165 patients were required in each cohort. Detection rate per patient was additionally tested in a right-sided binominal test with the alternative hypothesis that the proportion of successful SLNBs would be >0.93 for each tracer. A *p*-value of <0.05 would indicate that the null hypothesis was rejected. To allow for direct comparisons and to define factors affecting outcomes, patient-level data from the Nordic trial [13] were used as a third, reference cohort and comparisons were performed as a one-step individual patient data (IPD) meta-analysis [21].

Demographic and clinical patient data, tumor characteristics, intraoperative magnetic and radioisotope signals, SLN-specific data, tracer-specific data, pre/postoperative histopathological data, possible adverse events and postoperative staining were recorded.

The primary endpoint was the proportion of successful magnetic SLN procedures divided by the total number of SLN procedures performed (detection rate per patient). A procedure was defined as successful for the respective tracer if at least one SLN was identified and retrieved. Secondary endpoints were (a) nodal detection rate, defined as the number of magnetic SLNs identified, divided with the number of SLNs detected with both modalities, (b) the average number of excised SLNs per patient, (c) the proportion of pathologically positive SLNs per patient and per node (malignancy rate) and (d) the SPIO-RI SLN concordance rate per patient and per node, defined as the proportion of patients or nodes detected by both SPIO and RI to the patients or nodes detected by RI.

All endpoints were analyzed at two different cut-off points with regards to the Sentimag^®^ signal of the SLN, >0 and >20. The latter was selected to adjust for overlapping of detection methods (RI vs. SPIO), as nodes with low signal on one probe and high on the other, while formally considered as SLNs detected with both methods, would probably not have been identified had the patient received only one tracer.

The manuscript was prepared according to the “Strengthening the Reporting of Observational Studies in Epidemiology” (STROBE) statement [22], and the Preferred Reporting Items for Systematic Review and Meta-Analyses of individual participant data (the PRISMA-IPD) statement was followed for database formation and statistical analyses [23]. Subsequently, any differences in study design or inclusion criteria between the SentiDose protocol and the Nordic trial protocol were parametrized as independent input variables, to allow for harmonization of definitions and the conduct of multivariable regression analyses, as appropriate. This resulted in an individual patient-level dataset comprising of the Nordic trial population (retroareolar/interstitial injection of 2 mL Sienna+^®^ (Endomagnetics Ltd.) diluted with 3 mL of NaCl 0.9% or local anesthetic, administered perioperatively) used as a historic reference and the two prospectively collected cohorts of the SentiDose trial, as described above.

Comparisons of numeric outcomes were performed by one-way analysis of variance (ANOVA), whereas dichotomous outcomes were analyzed by means of Pearson’s *χ*^2^. Bonferroni adjustment for multiple comparisons was performed. Multivariable regression was performed if univariable associations with *p* < 0.1 were detected among clinically relevant variables. Background within-patient comparisons between SPIO and RI ± BD were performed to ensure non-inferiority and patient safety, but were not intended in the statistical analysis plan and thus, the published endpoints of the Nordic trial were not repeated.

### 2.3. Staining

All patients were prospectively followed for postoperative skin staining by SPIO or BD. Herein, patients with a brown/grey skin discoloration up to 6 months post-surgery were recorded. Long-term follow-up will be reported elsewhere.

## 3. Results

Consecutive patients were recruited, with the 1.5 mL cohort (*n* = 165) completed between August 2017 and April 2018 and the 1.0 mL cohort (*n* = 165) between May 2018 and September 2019. Protocol violation led to the exclusion of two patients from the 1.5 mL cohort. In total, 534 patients were analyzed and their characteristics are described in Table 1. There were no significant differences between the cohorts with regards to age, body mass index (BMI), tumor size, tumor type, tumor biology, or the proportion of patients with SLN metastasis. The SPIO injections were well-tolerated and no adverse effects were reported in the groups.

### 3.1. Sentinel Lymph Node Identification—Per Patient

The overall magnetic SLN detection rate per patient was 97.6% in the Nordic trial, 97.5% in the 1.5 mL cohort and 100% in the 1.0 mL cohort (*p* = 0.110). Multivariable regression analysis showed a trend for significance for previous breast surgery with regards to the per-patient SLN magnetic detection rate at >0 magnetic tracer signal cut-off (b = 5.435, 95% confidence interval (CI) 0.925, 31.935; *p* = 0.061), and significance for previous breast surgery at >20 cut-off (b = 6.957, 95% CI 1.552, 31.192, *p* = 0.011). The detection rate of pathologically positive SLNs (malignancy rate) was 96.3% in the Nordic trial, 97% in the 1.5 mL cohort and 100% in the 1.0 mL cohort (*p* = 0.796). The SPIO-RI concordance rates were 98% vs. 97.8% vs. 100%, respectively (*p* = 0.115). The concordance rate with regards to patients with pathologically positive SLNs was 98% in the Nordic trial, 97% in the 1.5 mL cohort and 100% in the 1.0 mL cohort (*p* = 1.0) (see Table 2).

### 3.2. Sentinel Lymph Node Identification—Per Node

The nodal detection rate was 93.3% in the Nordic trial, 85.6% in the 1.5 mL cohort and 97% in the 1.0 mL cohort (*p* = <0.001). The mean number of SLNs retrieved in the three cohorts was 1.83 vs. 1.85 vs. 2.18 (*p* = 0.003). The SPIO malignancy rate per node was 93.8% in the Nordic trial, 79.5% in the 1.5 mL cohort and 100% in the 1.0 mL cohort. In multivariable analysis, preoperative injection (1–7 days) was associated with the retrieval of more SLNs and a higher nodal detection rate. Detailed per-node results are reported in Table 3 and Table 4.

### 3.3. Effect of Injection Site and Injection Timing on SLN Detection

For a magnetic signal > 0, SLN detection after a periareolar injection was 97.9% vs. 100% after a peritumoral injection (*p* = 0.301), and for a magnetic signal > 20, 96.9% vs. 100%, respectively (*p* = 0.174). Regarding injection timing, a preoperative injection (1 to 7 days before surgery) was found to enhance SLN for a magnetic signal > 0 (100% vs. 97.6% for perioperative injection, *p* = 0.063). Looking into magnetic signal > 20, the difference was larger in favor of preoperative injection (100% vs. 96.5%, *p* = 0.012). This difference was retained in multivariable logistic regression. Regarding the number of SLNs retrieved, multivariable linear regression showed that periareolar injection was linked with a trend of retrieving less SLNs (b = 0.215, 95% CI −0.036, 0.465, *p* = 0.093), but the result was not statistically significant.

### 3.4. Skin Staining

The incidence and size of SPIO staining at 6 months in women undergoing breast conserving therapy (BCT) were not significantly different between the 1.5 mL cohort and the 1.0 mL cohort: 25.6% (33/129) vs. 18.4% (26/141) (*p* = 0.15), with mean sizes of 13.4 and 11.2 cm^2^ (*p* = 0.16). In multivariable logistic regression, a peritumoral injection was associated with less skin staining.

## 4. Discussion

In the largest patient dataset to date, lowering SPIO volume to 1.0–1.5 mL did not affect SLN detection. The SLN detection rate per patient was at least 96.7%, constantly comparable to RI ± BD and unaffected by SPIO dose, timeframe and injection site. Moreover, different doses, injection timeframes and sites resulted in equally high SPIO-RI concordance rates.

These findings are consistent with recent results by Alvarado et al. [19] and Rubio et al. [20]. In these studies, however, SPIO was administered intraoperatively and injected in the subareolar area. The present results provide more evidence that, not only can a smaller dose be equally efficient, but also that an extended injection timeframe in the preoperative period might enhance the detection rate and SLN retrieval. It seems that preoperative injection allows for higher SPIO concentration in the SLN, which was demonstrated in the present study by the fact that there were no “low-signal” SLNs in this patient group and that brown coloring of the SLN was more intense. In addition, not only were there more SLNs retrieved, but the nodal detection rate was also higher, indicating that preoperative SPIO injection allows for accumulation in the SLNs, whereas SPIO in the lymphatics, which may produce a “magnetic background”, is washed away to the circulation. Whilst the mean number of SLNs retrieved in patients injected preoperatively was 2.2, SPIO-RI nodal concordance was as high as 97%, demonstrating that there is no risk that the magnetic tracer would yield an unnecessary increase in the mean number of SLNs excised.

In previous studies from our group, results have shown that a preoperative injection of SPIO can be extended to more than 30 days before surgery with equally high SLN detection rates [14,17,24], but this had not been tested with a reduced SPIO dose. It is now clearer that timeframe is probably more important than the dose itself. In this context, SPIO is a highly effective tracer because it yields very high detection rates, but at the same time provides flexibility and ease of administration, as it can be injected both intraoperatively and also at the outpatient clinic, sparing intraoperative time and resources and facilitating logistics.

Skin staining after SPIO injection is a concern, although several reports have shown that most patients do not consider it a problem [14,17,20]. In the SentiMagIC study [19], skin discoloration after a 2.0 mL subareolar injection was reported in 15.6% of patients. However, the proportion of BCS and the time for follow-up were not specified.

In the SUNRISE study by Rubio et al. [20], using subareolar injections in patients who underwent BCT resulted in staining varying from 59% in patients who received 1.0 mL to 83.3% in patients who received 2.0 mL. In the present results, a deeper, peritumoral injection seems to be associated with less skin staining, consistent with previous findings [14,17], implying that excision of the SPIO-stained injection site reduces the skin staining rate. A peritumoral injection and a smaller SPIO dose might also address the concern that has been reported for postoperative magnetic resonance imaging (MRI) artifacts [25], as the bulk of SPIO is excised with the tumor. Currently, our group is accruing data to specifically address this issue within the prospective POSTMAG MRI trial [26]. Despite that flexibility in injection site in the 1.0 mL cohort may have not allowed for the formation of two patient cohorts with distinctive characteristics, the study protocol allowed flexibility in the second cohort regarding the injection site, as manufacturer instructions during the study period stated that periareolar injection can be applied intraoperatively, regardless of dose, but peritumoral might require a longer time. At the same time, analysis of other data from our group published elsewhere [17] were in favor of a deeper injection, achieving comparable detection rates and resulting in less skin staining. Those previous conclusions are confirmed in the present results.

The study design did not include patient randomization, which is the standard robust methodological approach [27]. However, given the fact that study participants stemmed from the same reference population and that no differences in baseline patient demographics or tumor data could be demonstrated, implementing randomization would have been highly challenging for logistics in the multicenter setting without necessarily adding much more to the study results [28]. The technique of one-stage IPD meta-analysis was utilized, so as to improve the quality of data and expand the type of analyses that may be performed, thus producing more reliable results than the comparison with aggregate or historical data [29]. In the particular dataset, the homogeneity of study populations and protocols between the Nordic trial and the SentiDose suggests low risk of ecological bias, and within- and across-studies information do not differ substantially [30]. This resulted in a large patient dataset, highly representative of the relevant background population of breast cancer patients. Additionally, the study was performed in diverse clinical settings, including both university and regional hospitals, and breast cancer units that use SPIO routinely or not. This fact reflects a pragmatic value to the applicability of the study results, as they reflect routine practice rather than highly selected cases of patients. On the other hand, less exclusion criteria might have added more to study pragmatism, but that would have, in turn, created more patient subgroups and deviated from the primary aim of the trial, which was to investigate the performance of lower SPIO doses.

In a large patient dataset, it is now shown that a reduction down to half of the stipulated dose is highly effective and that a deeper preoperative injection yields more SLNs while retaining a high SPIO-RI concordance rate and resulting in less skin staining, when injected peritumorally. The use of SPIO in other clinical situations, such as SLN identification and dissection in malignant melanoma [31], prostate cancer [32,33,34], penile cancer [35] and uterine cancer [36], has been investigated, with interesting implementations.

Regarding breast cancer, the present results build on a substantial body of evidence that renders SPIO a very effective SLN tracer, that should not be considered an alternative to the RI anymore, as the comparable performance, ease of access and flexibility in delivery of care are important properties for clinical routine and implementation in the global setting. In this context, long-term follow-up and more studies to address specific clinical situations is paramount, in order to reach a robust and clinically relevant conclusion.

## 5. Conclusions

Magtrace^®^ in lower doses (1.5 mL, 1.0 mL) is noninferior for SLN detection in patients with breast cancer compared with Sienna+^®^ and highly concordant with the dual technique. Apart from perioperative administration, it was shown that preoperative peritumoral injection of 1.0 mL not only facilitated logistics but also increased detection rate and nodal yield, with high concordance with the dual technique with the additional advantage of less skin staining. Magnetic-guided SLN detection not only has the potential to omit isotope-based axillary mapping but preoperative administration allows for novel implementations to meet tailored needs of breast cancer patients.

## Figures and Tables

**Table 1:** Patient and tumor characteristics.

		Nordic Trial (2 mL)	Sentidose Trial (1.5 mL)	Sentidose Trial (1.0 mL)	*p*-Value
Patients, *n* = 534		206	163	165	n.a.
Age, years (mean)		62	64	63	0.101 *
BMI, kg/m^2^ (mean)		27.9	27.2	26.5	0.568 *
Tumor size, mm (mean)		19	20	20	0.751 *
Histology	DCIS	12	9	4	0.694 ^#^
	IDC	158	122	121	
	ILC	26	21	28	
	Other	10	11	12	
ER-status	Positive	170	138	145	0.831 ^#^
	Negative	20	13	16	
	Missing	16	12	4	
HER2-status	Positive	20	9	16	0.217 ^#^
	Negative	172	142	144	
	Missing	14	12	5	
Ki67 (%) (mean)		26.6	23.5	25.2	0.349 *
No. patients with metastasis		54	33	29	0.120 ^#^
Previous ipsilateral breast surgery	Yes	17	0	0	<0.001 ^#^
	No	189	163	165	
Previous ipsilateral axillary surgery	Yes	3	0	0	0.114 ^#^
	No	203	163	165	
Type of surgery	BCT	154	130	141	0.038 ^#^
	Mastectomy	52	33	24	
SPIO Injection site	Peri-/Sub-areolar	198	157	68	<0.001 ^#^
	Peritumoral	3	6	97	
	Missing	5	0	0	

BCT: breast conserving therapy, BMI: body mass index, DCIS: ductal cancer in situ, ER: estrogen receptor, HER2: Human epithelial growth factor receptor type 2, IDC: invasive ductal cancer, ILC: invasive lobular cancer, n.a.: not assessed, SPIO: superparamagnetic iron oxide. *: analysis of variance (ANOVA), ^#^: Pearson’s *χ*^2^ test.

**Table 2:** Sentinel lymph node identification—per patient.

*n* = 534	Nordic Trial (2 mL) *n* = 206	Sentidose Trial (1.5 mL) *n* = 163	Sentidose Trial (1.0 mL) *n* = 165	*p*-Value
SPIO SLN detection rate (%)				
If magnetic signal > 0	97.6	97.5	100	0.110 *
If magnetic signal > 20	97.1	95.7	100	0.016 *
SPIO SLN detection rate, malignancy (%)				
If magnetic signal > 0	96.3	97.0	100	0.796 ^#^
If magnetic signal > 20	94.4	97.0	100	0.693 ^#^
SPIO-RI SLN concordance (%)				
If magnetic signal > 0	98.0	97.8	100	0.115 ^#^
If magnetic signal > 20	97.5	93.8	100	0.265 ^#^
SPIO-RI SLN concordance, malignancy (%)				
If magnetic signal > 0	98.1	97	100	1.000 ^#^
If magnetic signal > 20	96.2	100	100	1.000 ^#^

^#^: Pearson’s *χ*^2^ test. * Detection rates compared with Fisher’s exact test. Concordance calculated on cross-tabulations with use of the McNemar’s test. SPIO: superparamagnetic iron oxide nanoparticles, SLN: sentinel lymph node, RI: radioisotope.

**Table 3:** Sentinel lymph node identification—per node.

*n* = 534	Nordic Trial (2 mL) *n* = 206	Sentidose Trial (1.5 mL) *n* = 163	Sentidose Trial (1.0 mL) *n* = 165	*p*-Value
No. SPIO SLNs (mean)				
If magnetic signal > 0	1.83	1.85	2.18	0.003 *
If magnetic signal > 20	1.80	1.83	2.18	0.016 *
Nodal detection rate (%)				
If magnetic signal > 0	93.3	85.6	97	<0.001 ^#^
If magnetic signal > 20	92	84.9	97	<0.001 ^#^
No. SPIO SLNs, malignancy (mean)				
If magnetic signal > 0	1.11	0.8	1.18	<0.001 *
If magnetic signal > 20	1.11	0.8	1.18	<0.001 *
Nodal detection rate, malignancy (%)				
If magnetic signal > 0	93.8	79.5	100	0.005 ^#^
If magnetic signal > 20	93.8	79.5	100	0.005 ^#^
Nodal SPIO-RI concordance (%)				
If magnetic signal > 0	92.3	87.6	97.1	<0.001 ^#^
If magnetic signal > 20	100	87.2	96.8	<0.001 ^#^
Nodal SPIO-RI concordance, malignancy (%)				
If magnetic signal > 0	96.3	79.4	100	0.009 ^#^
If magnetic signal > 20	100	74.4	100	<0.001 ^#^

*: ANOVA, ^#^: Pearson’s *χ*^2^ test.

**Table 4:** Cross-tabulation—Sentinel lymph node detection—in total numbers/cohort.

**Nordic Trial Cohort**									
**Magnetic signal > 0**					**Magnetic signal > 20**				
		Radioisotope					Radioisotope		
		Yes	No	Total			Yes	No	Total
SPIO	Yes	368	8	376	SPIO	Yes	323	48	371
	No	6	22	26		No	27	4	31
	Total	372	30	402		Total	350	52	402
**SentiDose 1.5 mL cohort**									
**Magnetic signal > 0**					**Magnetic signal > 20**				
		Radioisotope					Radioisotope		
		Yes	No	Total			Yes	No	Total
SPIO	Yes	298	0	298	SPIO	Yes	275	26	301
	No	6	47	53		No	38	12	50
	Total	304	47	351		Total	313	38	351
**SentiDose 1.0 mL cohort**									
**Magnetic signal > 0**					**Magnetic signal > 20**				
		Radioisotope					Radioisotope		
		Yes	No	Total			Yes	No	Total
SPIO	Yes	300	59	359	SPIO	Yes	299	61	360
	No	9	3	12		No	10	1	11
	Total	309	62	371		Total	309	62	371

## Data Availability

The data presented in this study are available on request from the corresponding author. The data are not publicly available due to ethical considerations and data regulations.
